# Supplementary material for: Eco-Friendly Dispersant-Free Purification Method of Boron Nitride Nanotubes through Controlling Surface Tension and Steric Repulsion with Solvents
Source: Nanomaterials (Basel). 2023 Sep 19;13(18):2593. doi: 10.3390/nano13182593 (PMC10537017; doi:10.3390/nano13182593)
Supplement: Supplementary file 1 [file nanomaterials-13-02593-s001.zip › nanomaterials-2623800-supplementary.pdf]

## Supplementary Materials

# Eco-Friendly Dispersant-Free Purification Method of Boron Nitride Nanotubes through Controlling Surface Tension and Steric Repulsion with Solvents

**Minsung Kang [1,2,†], Jungmo Kim [3,†], Hongjin Lim [1,†], Jaehyoung Ko [1], Hong-Sik Kim [1], Yongho Joo [1], Se Youn Moon [4], Se Gyu Jang [1], Eunji Lee [2,*] and Seokhoon Ahn [1,*]**

1 Institute of Advanced Composite Materials, Korea Institute of Science and Technology (KIST), Jeonbuk 55324, Republic of Korea; t19132@kist.re.kr (M.K.)
2 School of Materials Science and Engineering, Gwangju Institute of Science and Technology (GIST), Gwangju 61005, Republic of Korea
3 Nano Hybrid Technology Research Center, Korea Electrotechnology Research Institute (KERI), Changwon-si 51543, Republic of Korea; jungmokim@keri.re.kr
4 Department of Quantum System Engineering, Jeonbuk National University, Jeonju-si 54896, Republic of Korea; symoon@jbnu.ac.kr
* Correspondence: eunjilee@gist.ac.kr (E.L.); ahn75@kist.re.kr (S.A.)
† These authors contributed equally to this work.

**Keywords:** BNNT; purification; eco-friendly; cosolvent; interfacial energy

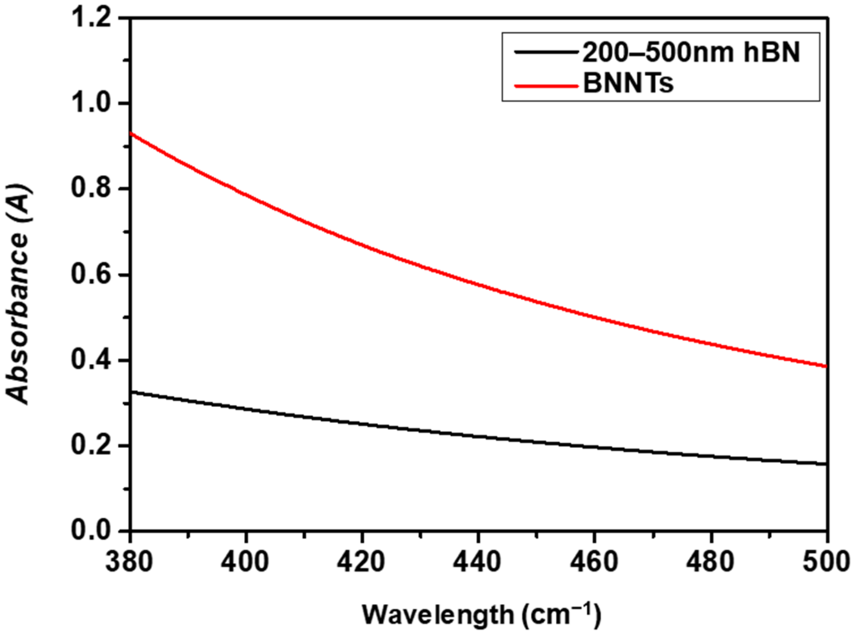


**Figure S1**. UV-NIR spectra of BNNTs and 200–500 nm hBN at 70 wt% tBA/DIW mixture.

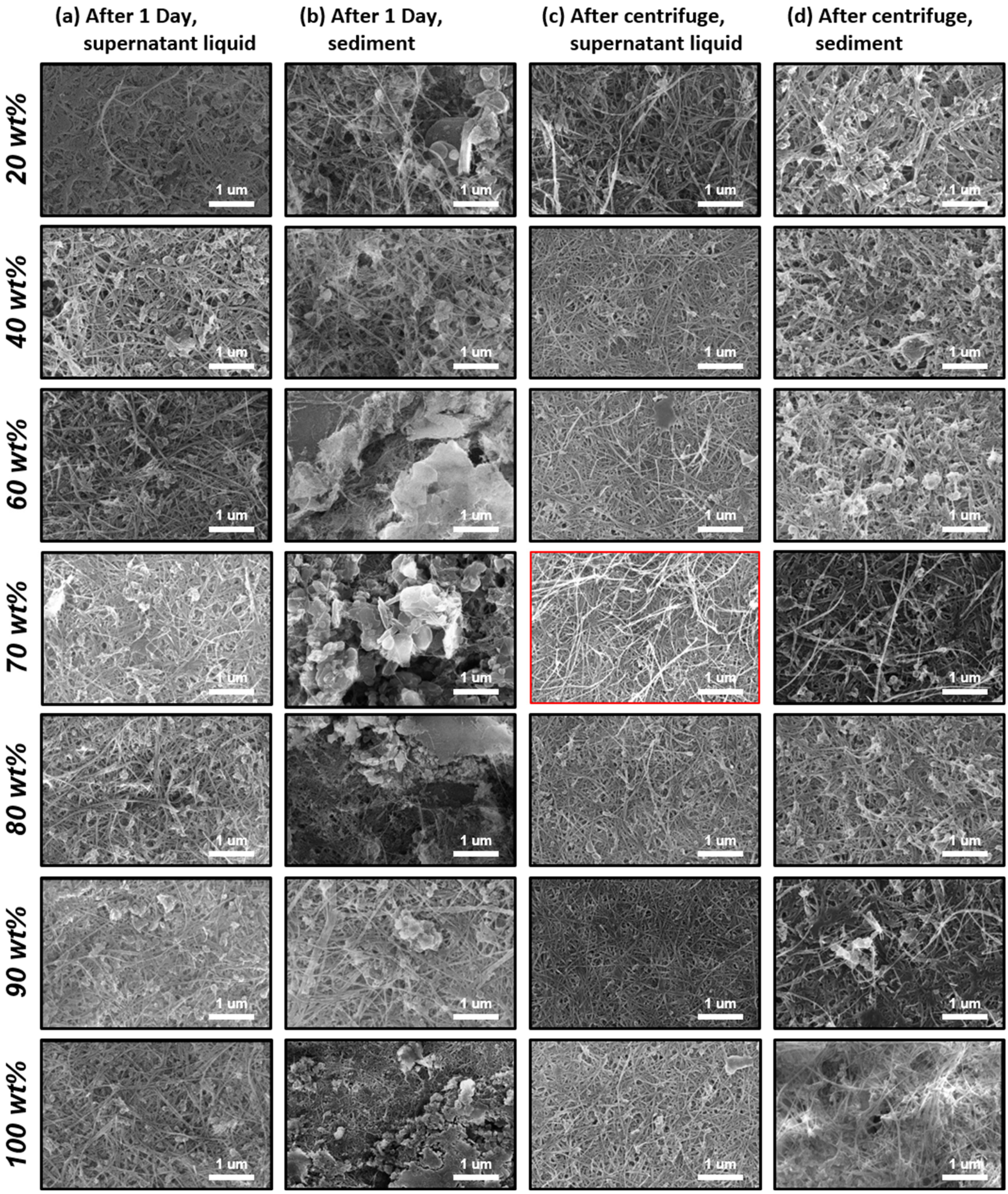


**Figure S2**. SEM image of supernatant (a), (c) and sediment (b), (d). Image of (a) and (b) are solutions 1 day after dispersion. Images of (c) and (d) are the results obtained after centrifugation from the (a) solution.

**Table S1.** Surface tension value of t-BA/DIW mixtures were obtained from the [1,2].

| Mass % | 0 % | 10 % | 20 % | 30 % | 40 % | 50 % | 60 % | 7 0% | 80 % | 90 % | 100 % |
|---|---|---|---|---|---|---|---|---|---|---|---|
| Surface tension (mN/m) | 72.0 | 32.7 | 25.6 | 23.7 | 23.2 | 22.6 | 22.0 | 20.9 | 20.4 | 20.1 | 20.1 |

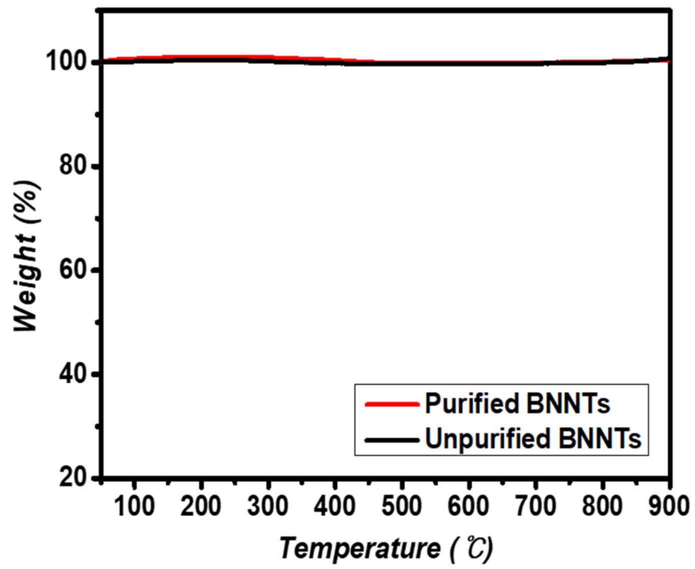


**Figure. S3**. TGA analysis data of unpurified and purified BNNTs showing no organic residues.

To determine the relative purification efficiency of the system, the following equation was used [3].

$$\textit{Relative hBN removal effieciency (\%)} = 100 - \left(\frac{A_{hBN,\,sample} \times \frac{A_{BNNTs,\,crude}}{A_{BNNTs,\,sample}}}{A_{hBN,\,crude}}\right) \times 100$$

In above the equation, A is the area of the deconvoluted peak. **Figure. S4** illustrates the differences in the XRD patterns of the unpurified BNNTs (as-synthesized BNNTs, calcined BNNTs, and $B_2O_3$ removed BNNTs) and the purified BNNTs (at 5000 rpm and 10,000 rpm). The major peak in as-synthesized BNNTs corresponded to hBN, $B_2O_3$, and a-B peak at 2Θ = 26.8°, 27.95°and 25.1°, respectively [4-8]. It was confirmed that these peaks of a-B and $B_2O_3$ in XRD pattern of the as-synthesized-BNNTs and calcined BNNTs, could be easily removed through calcination & hot-deionized water-washing process. In the $B_2O_3$ removed-BNNTs, a low intensity BNNTs peak (2θ = 25.8°) was detected due to the hBN peak. Because, this peak, which consists of 2D materials such as graphene and hBN, was very strong and sharp, while 1D materials such as BNNTs were weak and broad, relatively [4]. **Table S1** showed the deconvoluted patterns and calculated values by applying Gaussian fitting to the XRD patterns of three samples ($B_2O_3$ removed BNNTs and the purified BNNTs at 5000 rpm and 10,000 rpm).

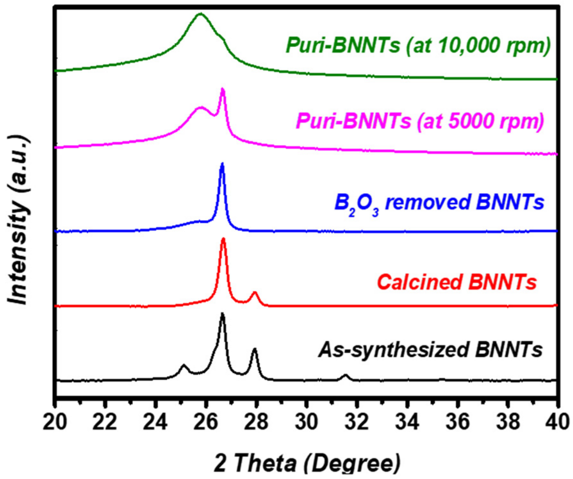


**Figure. S4**. The change of XRD pattern depending on purification process.

**Table S2.** Deconvolution of XRD pattern and calculated value for (a) $B_2O_3$ removed, (b) Purified BNNTs at 5000 rpm, and (c) Purified BNNTs at 10,000 rpm

| | **(a)** | | **(b)** | | **(c)** | |
|---|---|---|---|---|---|---|
| **Model** | Gauss | | Gauss | | Gauss | |
| **Equation** | $y=y_0+(A/w\times sqrt(PI/2))) \times exp(-2\times ((x-x_c)/w)^2)$ | | $y=y_0+(A/w\times sqrt(PI/2))) \times exp(-2\times ((x-x_c)/w)^2)$ | | $y=y_0+(A/w\times sqrt(PI/2))) \times exp(-2\times ((x-x_c)/w)^2)$ | |
| **COD($R^2$) / Adj. R-Square** | 0.99096 / 0.99068 | | 0.99751 / 0.99744 | | 0.99880 / 0.99876 | |
| **Plot** | Peak 1(BNNTs) | Peak 2(hBN) | Peak 1(BNNTs) | Peak 2(hBN) | Peak 1(BNNTs) | Peak 2(hBN) |
| **$Y_0$** | 5.1516 ± 0.12 | 5.1516±0.12 | 0.9217±0.085 | 0.9217±0.085 | 1.2716±0.080 | 1.2716±0.080 |
| **$x_c$** | 26.0617±0.023 | 26.676±0.0011 | 25.866±0.0046 | 26.667±0.0011 | 25.833±0.00252 | 26.696±0.0054 |
| **W** | 1.865±0.045 | 0.1956±0.0025 | 1.0912±0.0097 | 0.230±0.0026 | 1.0657±0.0055 | 0.288±0.014 |
| **A** | 28.932±0.85 | 20.182±0.26 | 73.174±0.61 | 22.516±0.31 | 128.479±0.60 | 5.979±0.36 |

## References


1. Gliński, J.; Chavepeyer, G.; Platten, J.K. Surface properties of diluted aqueous solutions of tert‐butyl alcohol. *J. Chem. Phys.* **1995**, *102*, 2113-2117, doi:https://doi.org/10.1063/1.468733.
2. Marsh, K.L.; Souliman, M.; Kaner, R.B. Co-solvent exfoliation and suspension of hexagonal boron nitride. *Chem Commun (Camb)* **2015**, *51*, 187-190, doi:https://doi.org/10.1039/C4CC07324J.
3. Ko, J.; Kim, D.; Sim, G.; Moon, S.Y.; Lee, S.S.; Jang, S.G.; Ahn, S.; Im, S.G.; Joo, Y. Scalable, Highly Pure, and Diameter-Sorted Boron Nitride Nanotube by Aqueous Polymer Two-Phase Extraction. *Small Methods* **2023**, *7*, 2201341, doi:https://doi.org/10.1002/smtd.202201341.
4. Amin, M.S.; Atwater, B.; Pike, R.D.; Williamson, K.E.; Kranbuehl, D.E.; Schniepp, H.C. High-Purity Boron Nitride Nanotubes via High-Yield Hydrocarbon Solvent Processing. *Chem. Mater.* **2019**, *31*, 8351-8357, doi:https://doi.org/10.1021/acs.chemmater.9b01713.
5. Balint, M.G.; Petrescu, M.I. An attempt to identify the presence of polytype stacking faults in hBN powders by means of X-ray diffraction. *Diam. Relat. Mater* **2009**, *18*, 1157-1162, doi:https://doi.org/10.1016/j.diamond.2009.02.035.
6. Harrison, H.; Lamb, J.T.; Nowlin, K.S.; Guenthner, A.J.; Ghiassi, K.B.; Kelkar, A.D.; Alston, J.R. Quantification of hexagonal boron nitride impurities in boron nitride nanotubes via FTIR spectroscopy. *Nanoscale Adv* **2019**, *1*, 1693-1701, doi:https://doi.org/10.1039/C8NA00251G.
7. Kumari, S.; Sharma, O.P.; Gusain, R.; Mungse, H.P.; Kukrety, A.; Kumar, N.; Sugimura, H.; Khatri, O.P. Alkyl-chain-grafted hexagonal boron nitride nanoplatelets as oil-dispersible additives for friction and wear reduction. *ACS Appl. Mater. Interfaces.* **2015**, *7*, 3708-3716, doi:https://doi.org/10.1021/am5083232.
8. Singh, D.K.; Iyer, P.K.; Giri, P.K. Diameter dependence of interwall separation and strain in multiwalled carbon nanotubes probed by X-ray diffraction and Raman scattering studies. *Diam. Relat. Mater* **2010**, *19*, 1281-1288, doi:https://doi.org/10.1016/j.diamond.2010.06.003.
